# Emergent Properties of Patch Shapes Affect Edge Permeability to Animals

**DOI:** 10.1371/journal.pone.0021886

**Published:** 2011-07-01

**Authors:** Vilis O. Nams

**Affiliations:** Department of Environmental Sciences, Nova Scotia Agricultural College, Truro, Canada, and School of Marine and Tropical Biology, James Cook University, Townsville, Australia; Vrije Universiteit, Netherlands

## Abstract

Animal travel between habitat patches affects populations, communities and ecosystems. There are three levels of organization of edge properties, and each of these can affect animals. At the lowest level are the different habitats on each side of an edge, then there is the edge itself, and finally, at the highest level of organization, is the geometry or structure of the edge. This study used computer simulations to (1) find out whether effects of edge shapes on animal behavior can arise as emergent properties solely due to reactions to edges in general, without the animals reacting to the shapes of the edges, and to (2) generate predictions to allow field and experimental studies to test mechanisms of edge shape response. Individual animals were modeled traveling inside a habitat patch that had different kinds of edge shapes (convex, concave and straight). When animals responded edges of patches, this created an emergent property of responding to the shape of the edge. The response was mostly to absolute width of the shapes, and not the narrowness of them. When animals were attracted to edges, then they tended to collect in convexities and disperse from concavities, and the opposite happened when animals avoided edges. Most of the responses occurred within a distance of 40% of the perceptual range from the tip of the shapes. Predictions were produced for directionality at various locations and combinations of treatments, to be used for testing edge behavior mechanisms. These results suggest that edge shapes tend to either concentrate or disperse animals, simply because the animals are either attracted to or avoid edges, with an effect as great as 3 times the normal density. Thus edge shape could affect processes like pollination, seed predation and dispersal and predator abundance.

## Introduction

Animal travel between habitat patches affects populations, communities and ecosystems. On a large scale this affects metapopulation dynamics, where extinction rates of subpopulations are crucially dependent on dispersal rates [1]. On a small scale this affects population dynamics, where foraging success [2] and predation [3] are affected by travel across patchy landscapes. Thus it is important to understand the process of traveling from one habitat patch to another. One can separate this process into two separate behavioral components: the action of crossing the edge and entering the matrix, and then traveling through the matrix. This paper will focus on the component of edge-crossing.

There are three levels of organization of edge properties, and each of these can affect animals. At the lowest level are the different habitats on each side of an edge, then at a higher level there is the edge itself, and finally, at the highest level of organization, is the geometry or structure of the edge - for example, perimeter∶area ratio, corridors and edge shapes (convexities vs. concavities). This paper will focus on the highest level of edge organization, edge geometry.

One fundamental and widespread type of edge geometry is edge shape. The idea that animals might be affected by edge shape was first suggested by Hardt and Forman [4], who found that woody colonization of revegetated mines was affected by the shape of the edge between grassland mine spoils and the surrounding forested habitat. Specifically, woody colonizers preferentially settled in grassland areas adjacent to edges that were concave into the forest. Hardt and Forman [4] hypothesized that this was a result of how animals responded to edge shape - that in grassland, for protection, browsing herbivores would concentrate where the edge intrudes into the forest. These herbivores would then disperse seeds of woody colonizers in their faeces. However, even though this hypothesis was proposed more than 20 years ago, and even though all edges have either convexities or concavities, there have been no studies on how animals react to the shape of habitat edges. We need theoretical studies to generate testable hypotheses for different mechanisms of response, we need field observational studies to find out what kinds of responses animals show to different edge shapes, and we need experimental studies to test the different mechanisms of edge shape effects.

Edge properties are important in two ways: ecologically, what is important is whether the edge property affects the animal's movement, but mechanistically, what is important is whether the animal responds behaviorally to that edge property. And these might not be the same, because an animal's behavior to an edge property at a lower organizational level might cause an emergence effect at a higher level. For example, at a lower level, most animals respond differently to different habitats. If an animal avoids a habitat, then the edge will affect that animal's movement, without the animal recognizing the edge itself. Similarly, at a higher level, sleepy orange (*Eurema nicippe)* butterflies selectively travel through habitat corridors. However this can be explained solely by how they respond to edges; the butterflies' attraction to edges results in them concentrating in corridors, without having to recognize that these are specific types of edge structures [5]. Thus in order to fully understand how edge properties affect animal movement we need to understand whether reactions to those properties cause emergent effects.

Thus the objectives of this study are to use computer simulations to: (1) find out whether effects of edge shapes on animal behavior can arise as emergent properties solely due to reactions to edges in general, without the animals reacting to the shapes of the edges; and (2) generate predictions to allow field and experimental studies to test mechanisms of edge shape response.

## Methods

The general approach was to model individual animals traveling inside a habitat patch that had different kinds of edge shapes. Sizes and shapes of edge types was varied, as was the behavior of the animal to the edge (attraction, avoidance or neutral). I then compared densities and directions of animals at various places near the edge, to see how these were affected by edge shape and animal edge behavior. Simulations were carried out in Mathematical 7.0 [6].

**Figure 1:**
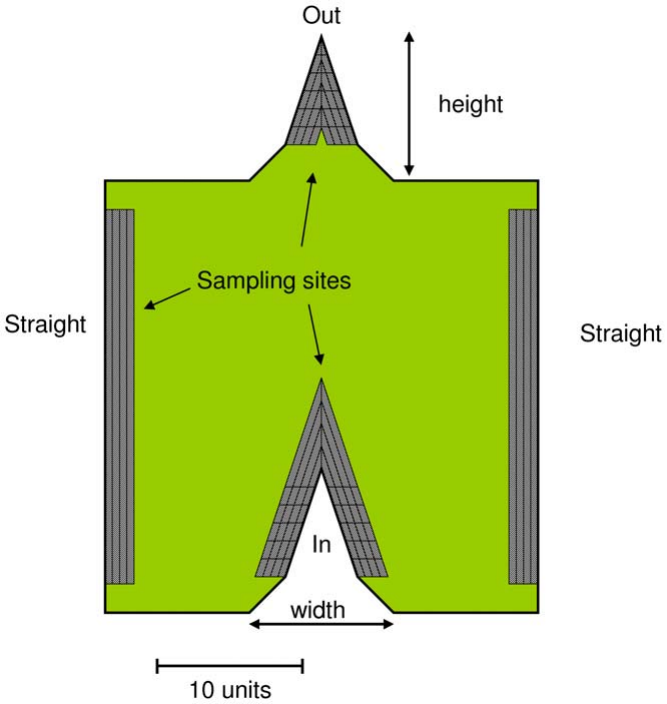
The simulated habitat patch that modeled animals traveled in. The grey areas show the sampling sites for the three different kinds of treatment edges. Size and shape of the edge types was varied.

Specifically, the habitat patch was 30×30 units in size (**Figure 1**) and contained three types of edge shapes: convex (called “In”), concave (called “Out”) and straight. The area outside of the patch was assumed to be inhospitable matrix, and thus animals did not leave the patch. The edge shapes were triangularly shaped in order to simplify comparisons, and the bases of the shapes were flared in order to minimize animal reactions to them. The dimensions were large enough to ensure that the edge types did not affect each other, and that the animal's behavior at the corners did not affect the edge shape responses; to ensure this, trial simulations were first run with different dimensions. Distance from corners to the Straight was 2 units, from a corner to the In and Out shapes was 10 units, between the In and Out shapes was 20 units, and point height was 10 units. These dimensions set the overall dimensions of the patch. I simulated all three types of edge shapes within one patch to make it easier to compare them.

I tested all combinations of 4 edge shape widths ×3 animal edge behaviors. Edge shape widths were 4, 7, 10, 15 units, and were measured at the width of the base. Animal edge behaviors were avoidance, neutral and attraction. Each run of the simulation used one type of avoidance behavior and was simulated by a run of 2,000,000 steps.

**Figure 2:**
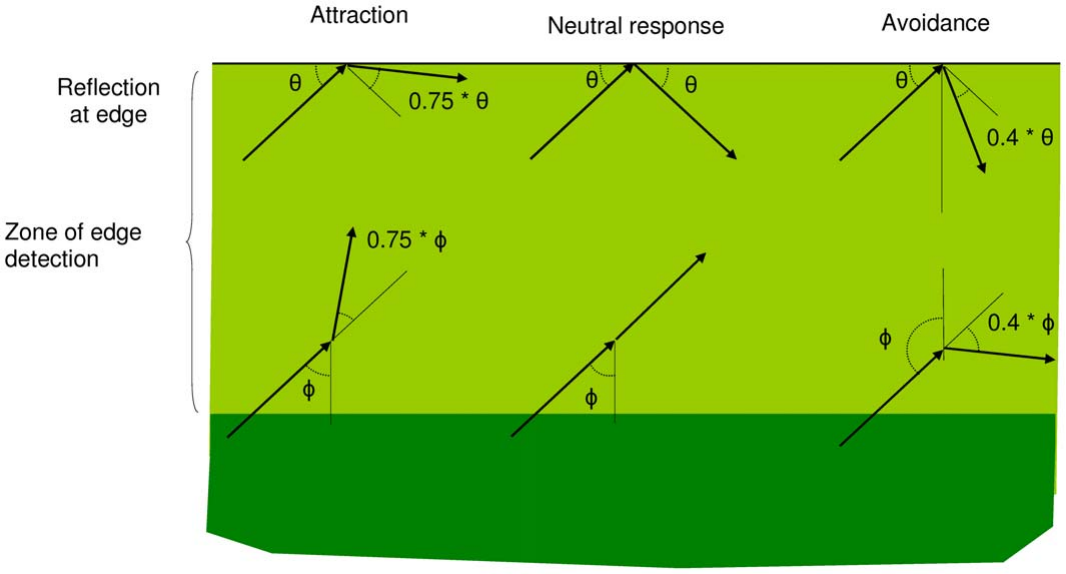
Three types of modeled behaviors to edges. The top row of figures represents how animals reflected from the edge and the bottom row represents how animals turned while within detecting distance of the edge.

The animal movement was simulated using a correlated random walk with independent turning angles distributed with a circular normal distribution [7], with a K-value of 5. Step length was fixed at one unit, and mean turning angle was zero when the animal was not near an edge. Edge behaviors were modeled by variations in mean turning angle. There were two regions where animals reacted to edges: when they detected the edge, and then once they actually intersected it. I modeled these decisions as follows (**Figure 2**). When the end of a step landed within the detection distance boundary, then the movement model changed to a biased correlated random walk [8], [9], with the following mean turning angles, 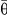: Neutral, 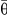 = 0; Attraction, 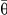 = 0.75 * (angle directly to the edge); Avoidance, 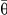 = 0.40 * (angle directly away from the edge).

**Figure 3:**
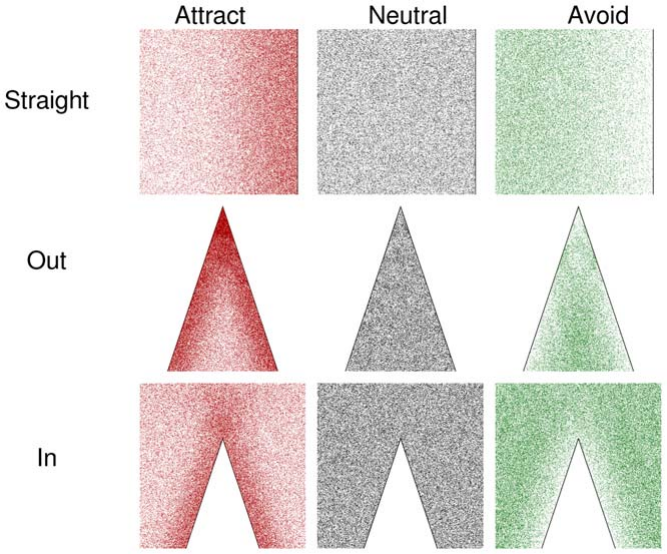
Density of animal locations vs. distance from edge of patch, for three different types of behaviors to the edge, for straight edges. Note the log scale for density. Densities are in proportion to the mean density in the whole patch - thus, 1 means no effect of the edge. Attraction and avoidance give proportionally similar effects, with most effects within 1 unit from the edge.

When animals intersected an edge, then their reflecting angles were affected by their edge behavior, with the following step angles, φ: Neutral, φ = mirror reflection angle; Attraction, φ = mirror reflection angle - 0.75 * (angle between mirror reflection and edge); Avoidance, φ = mirror reflection angle - 0.40* (angle between mirror reflection and perpendicular to edge). Note that after intersecting the edge, there was no random component to the new angle. The specific parameters were chosen so that attraction and avoidance would have a similar proportional effect on density - a 5x effect on density adjacent to the edge vs. inside (**Figure 3**).

Animal responses were measured at various positions along the edge and inside from the edge. Response statistics measured were density of locations and three measures of directionality. Densities were standardized by dividing by the mean density in the central part of the patch - thus a density of 1 means no edge effect.

I used three independent measures of directionality. The first measured edge-following - i.e. how parallel vs. perpendicular the trails were to the edge of the patch (called “Edge-following” from now on). Edge-following was estimated by |cos(θ)|−|sin(θ)|, where θ = the angle between the animal and the edge. Edge-following was −1 when the trail was perpendicular to the edge, 0 at 45°, and 1 when it was parallel to the edge.

The second one measured directionality towards the tip of the treatment, along the edge (called “To-tip” from now on). To-tip was estimated by sin(θ), where θ = the angle between the animal and the edge, oriented such that θ = 0° when pointing towards the edge. To-tip was 1 when the trail pointed towards the center of the treatment and -1 when it pointed away from the center. Thus 0 meant no directionality. To-tip was undefined for the Straight treatment.

The final one measured directionality towards the inside of the patch, perpendicular to the edge (called “To-inside” from now on). To-inside was estimated by cos(θ), where θ = the angle between the animal and the edge, oriented such that θ = 90° when the animal pointed towards the inside. To-inside was 1 when the trail pointed towards the mowed area and 1 when it pointed towards the outside. Thus 0 meant no directionality.

## Results

### Density

**Figure 4:**
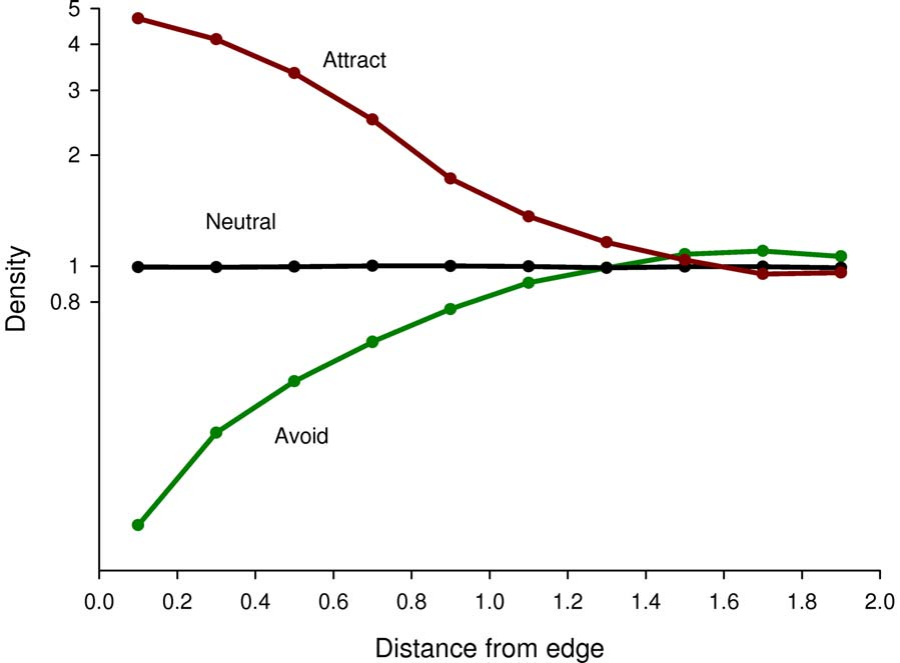
Example of densities of animals at different types of edge shapes, for width of shapes = 10.

Animal densities were obviously affected by edge shape (e.g. **Figure 4**). Densities were affected by the edge up to ∼1 unit away (**Figure 3**), so all of the rest of the results are presented for only 1 unit away from the edge.

**Figure 5:**
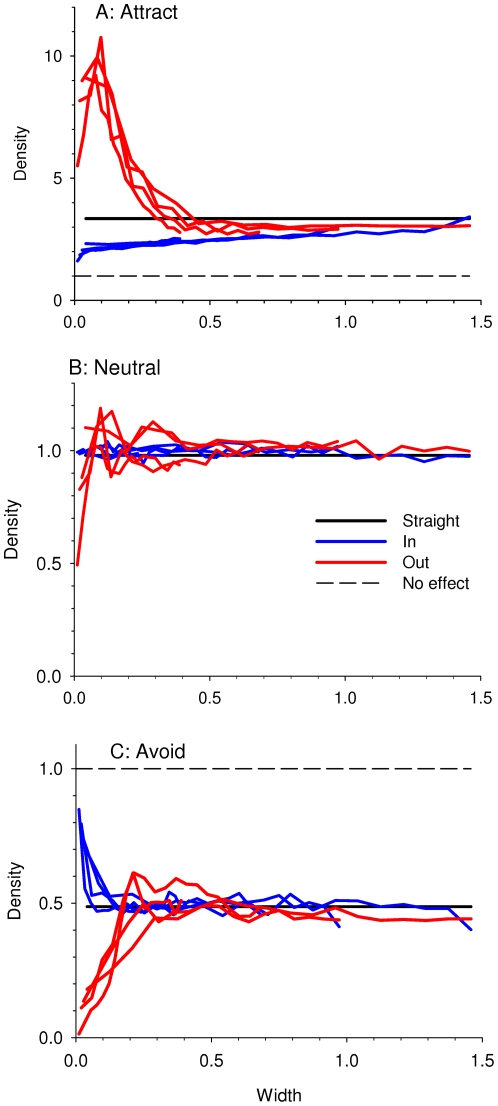
Density of animal locations vs. widths of edge shapes. Densities are in proportion to the mean density in the whole patch - thus, 1 means no effect of the edge. The three plots show different types of behaviors to the edge: **A** - attraction, **B** - neutral, **C** - avoidance. Within each graph, the different colors lines represent the different types of edge shapes. The different lines of each color represent different narrownesses. The tight clustering of the colored lines as compared to the change along the x-axis shows that most of the effects on density are due to point width, not point narrowness.

In order to compare the relative importance of absolute width vs. narrowness of the edge shapes, I combined all results into one plot (**Figure 5**), and plotted them vs. width. Each line joins all results from one narrowness. If animal effects chiefly varied according to narrowness of the shape, and not the absolute width, then we would expect that the lines would differ widely in their relationship to width. However if the opposite happened, that animal effects chiefly varied according to width, then we would expect that the lines would be quite similar in their relationship to width. Density effects are minimal for widths greater than ∼0.3–0.4 units for all measures of narrowness - even though narrowness varied 4-fold, from 0.4 to 1.5. Thus the absolute width of the shape is much more important than the narrowness, in the effects on density.

Comparing types of shapes, we see that animals were much more affected by the Out than the In shapes. For example, densities in the Out shape varied by a range of 100-fold when comparing Attraction to Avoidance (red lines in **Figure 5** **A** vs. **C**), whereas in the In shape they only varied by a range of 3-fold (blue lines in **Figure 5** **A** vs. **C**). Comparing animal edge behaviors, we see that the type of response depends on both shape and edge behavior. When animals are attracted to edges, then they collect more in Out and less in In than expected (red vs. blue in **Figure 5** **A**). The opposite happens when animals avoid edges (red vs. blue in **Figure 5** **C**). However, although they collect more in In shapes than Straight one when they avoid edges, they do still avoid In shapes overall (e.g. the maximum values are still <1).

### Directionality

**Figure 6:**
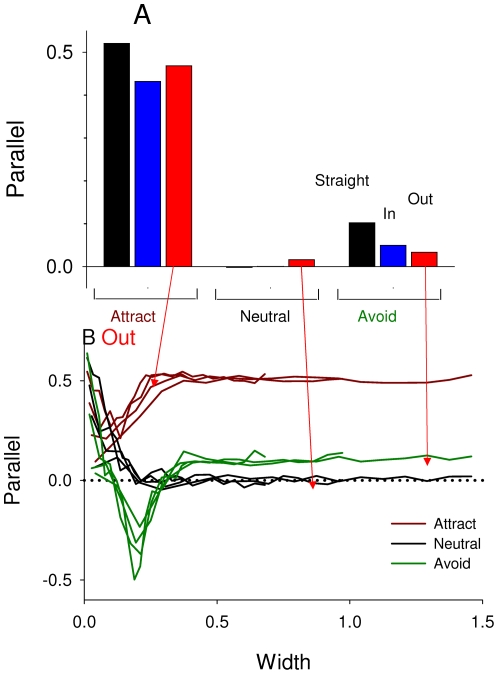
Edge-following, where 1 means completely parallel to the edge and -1 means completely perpendicular. **A**: Overall effects for different edge behaviors and edge shapes. Bars are grouped by behavior to edge, and within each group, bars show animals' behaviors to the edge: straight, in and out. The main differences are in the type of avoidance behavior, not in edge shapes. **B**: For the Out shape only, edge-following vs. width of shape. Each color represents different edge behaviors. With attraction (brown lines), animals lose directionality close to the tips of the shapes. With avoidance (green lines), animals orient perpendicular near the point, but then more parallel right at the point. With neutrality (black lines), animals orient parallel to the edge near the tips of the edge shapes.

For edge-following, the main differences are in the type of avoidance behavior, not in edge shapes (**Figure 6** **A**). When animals are attracted to the edge they tend to follow the edge, but when they are either neutral or avoid the edge, there is no clear parallel directionality. However, for the Out shapes, width has an effect on this relationships (**Figure 6** **B**). When animals are attracted to edges then they lose directionality close to the tips of the shapes. When animals are neutral or avoid edges, they travel quite parallel to the edge then near the tips of the shapes, but then this directionality drops off with edge width - and for avoidance, it goes to completely perpendicular at intermediate distances.

**Figure 7:**
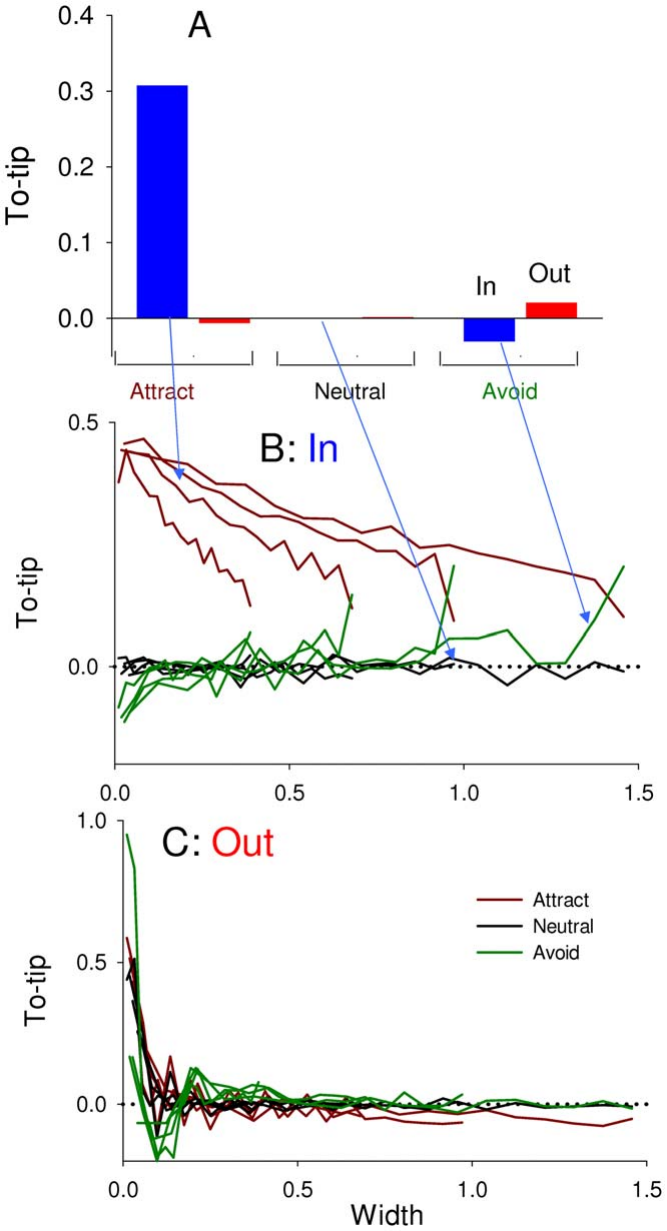
Directionality towards the tip of edge shapes, where 1 means pointed towards the tip of the shape along the edge, and -1 means pointed towards the base. **A**: Overall effects for different edge behaviors and edge shapes. Bars are grouped by behavior to edge, and within each group, bars show animals' behaviors to the edge: straight, in and out. There is no overall directionality except that when animals are attracted to edges, they orient towards the tips of In shapes. **B**: For the In shape only, To-tip vs. width of edge shape. Each color represents different edge behaviors. With attraction, animals orient more towards the tip the closer they are to the tip. **C**: For the Out shape only, To-tip vs. width of edge shape. With all behaviors, animals orient more towards the tip right at the tip of edge shapes.

Unlike edge-following, directionality towards the point shows a large difference within types of avoidance behaviors (**Figure 7** **A**). For all combinations there is no directionality, except that when animals are attracted to edges, they orient towards the point of In shapes, and with a decreasing directionality the further from the tip (**Figure 7** **B**). For all edge behaviors, the animals orient more towards the tips of Out shapes when closer to the tip (**Figure 7** **C**).

**Figure 8:**
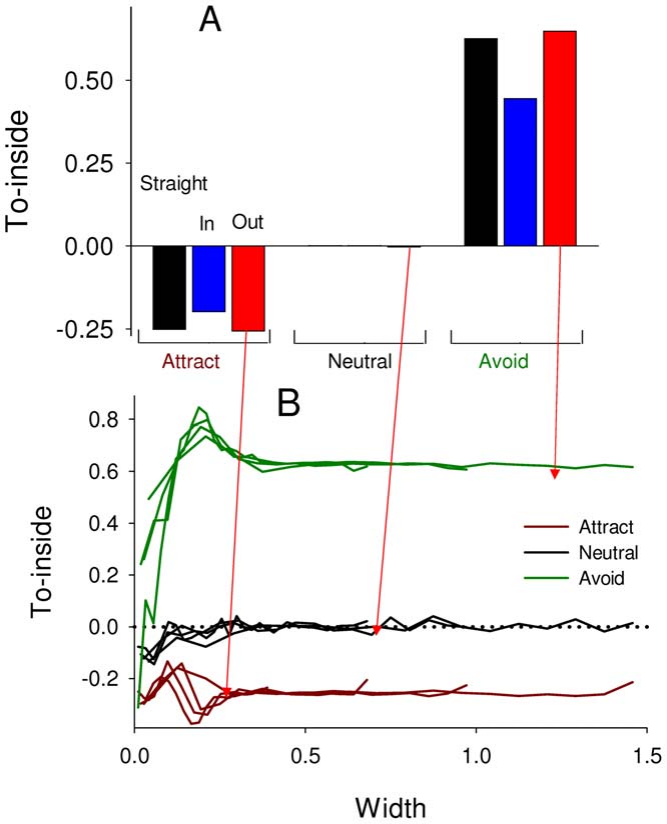
Directionality towards the inside, where 1 means pointed towards the inside of the patch, and -1 means pointed towards the outside. **A**: Overall effects for different edge behaviors and edge shapes. Bars are grouped by behavior to edge, and within each group, bars show animals' behaviors to the edge: straight, in and out. The main differences are in the type of avoidance behavior, not in edge shapes. **B**: For the Out shape only, To-inside vs. width of edge shape. Each color represents different edge behaviors. With all behaviors, directionality changes very close to the tips of the edge shapes.

Similarly to edge-following, for directionality towards the inside of the patch the main differences are in the type of avoidance behavior, not in edge shapes (**Figure 8** **A**). When animals are attracted to the edge then they tend orient to the outside, and when they avoid the edge then they orient to the inside. However this response decreases closer to the tips of the Out shapes (**Figure 8** **B**).

## Discussion

There are various assumptions this model makes which affect its application. The first assumption is that emigration rate depends on rates of encounters with patch boundaries - that organisms decide whether to emigrate or not, when they encounter the boundary. While this is also assumed by most theoretical models of metapopulation dynamics [10]–[12] it has never been explicitly tested. However there are some predictions of it that are supported. If emigration rate does depend on rates of encounters with patch boundaries, then we would expect that animals would emigrate more readily from patches with a greater perimeter:area ratio. This is seen with various butterfly species who immigrate out of small patches more (per area) than larger ones [13], [14], and ladybird beetles who emigrate from rectangular patches faster than square patches [15]. In fact, much of the observed variation in a series of published studies on emigration rate could be explained by the scaled effect of patch size [16]. On the other hand, this assumption is not globally valid, because a few studies have not found a relationship between emigration and patch shape [17]. Thus, the relation between edge encounter rate and emigration rate likely depends on the species.

The second assumption is about the realism of the algorithm of animal behavior at edges. It is difficult to assess this because little research has been done on how animals change movement in response to habitat edges. However, those few studies that measured relevant parameters do support my model. There are two important aspects to the edge-reaction model: the general structure and the specific parameters used.

The general structure has two parts to it - the reaction upon detecting the edge, and then after actually encountering it. In the first part, when my modeled animals detected an edge, they changed mean turning angle either towards or away from the edge by a specific bias value. A bias of 0 means a zero mean turning angle, a bias of 1 means the animal always turns directly towards the edge, and a bias of -1 means the animal always turns directly away from the edge. This type of behavior structure has been shown in the only two studies that have specifically measured turning angles near edges. Fender's blue butterflies (*Icaricia icarioides fenderi*) [8] and Eastern Bluebirds (*Sialia sialis*) [9] both are attracted to habitat edges and change their movement near edges by adding a bias to their turning angles.

In the second part of my edge reaction model, animals reflected from the edge with a variable angle. Here a bias of 0 means complete reflection, a bias of 1 means always parallel, and a bias of −1 means always perpendicular. No study has measured specific angles of reflection at edges, however, some have reported general agreement: chickadees (*Poecile atricapillus*) tend to travel parallel to edges [18] and that Eastern Bluebirds tend to travel either parallel or perpendicular to them [9]. Thus there is support for the biological realism of the structure of my edge-reaction model. However it would be useful for future theoretical models to test other aspects of edge behavior, such as the effects of animal behavior after it crosses the edge.

The specific parameters in my model for avoidance vs. attraction were chosen so that (1) attraction and avoidance gave proportionally similar, but opposite directions, responses to each other (e.g. **Figure 3**), and (2) that these would be extremes - that most species might be expected to show reactions within these ranges. For edge attraction, all published studies show bias effects less than the 0.75 used in my model. Fender's blue butterflies were attracted to edges with a bias of 0.09–0.61 for females and 0.22–0.38 for males [8], and Eastern Bluebirds were attracted with a bias of 0.74 [9]. For edge avoidance, no studies have measured the amount of edge bias. However two studies report general results which can be compared with mine. Ries and Debinski [19] measured the proportion of Regal fritillaries (*Speyeria idalia* Drury) and monarch (*Danaus plexippus* L.) butterflies turning away from habitat edges for 4 types of habitat edges. Six out of those eight combinations show a smaller edge avoidance than I observed (**Figure 3**). Ross et al. [20] measured the tendency for Rocky Mountain parnassian (*Parnassius smintheus)* butterflies to fly towards vs. away from forest edges at different distances from the edge. My measure of this tendency (variable To-inside, **Figure 8** **A**) showed an overall directionality towards the edge, averaged over all distances, which is reached by parnassian butterflies only within 0.3 units of the edge (**Figure 4** [20]). Thus the parameters I used for avoidance vs. attraction bounded the expected biological range.

### Predictions

Although my model only predicts edge shape effects when animals just respond to edges, in some situations we can use these predictions to test whether animals do recognize edge shapes. For example, Hardt and Forman [4] found that woody colonizers were more abundant in grassland areas adjacent to edges that were concave into the forest. Hardt and Forman [4] hypothesized that this happened because herbivores that typically live in the forests go into the grassy areas to feed, but that for protection, the herbivores would concentrate in the grassy areas where the edge intrudes into the forest – and thus disperse seeds of the woody colonizers into the grassland areas close to the concave edges. Thus their hypothesis is that herbivores specifically recognize and respond to edge shapes.

An alternate hypothesis, suggested by my model, is that herbivore behaviour at edge shapes is governed just by their response just to edges, not to edge shapes. First we need to determine their response to edges. Since the herbivores, that normally live in the forest, do graze on the grass in the open areas, this suggests that they are attracted to the edges. Then my model predicts that if they are attracted to edges, they would concentrate at convexities, not concavities. Thus we would expect that seed dispersal of the woody colonizers, and thus their density, would be greater at convexities.

Since my model's predictions are the opposite of Hardt and Forman's [4] results, this rejects the hypothesis that only edge response is important, and supports Hardt and Forman's [4] hypothesis that the herbivores actually recognize and respond to edge shapes. In this way, even though my model only addresses one mechanism of edge shape response (edge effects), one can use it sometimes to test for edge shape recognition.

Another aspect of predictions is the scale of the edge shapes. The units used in this model are biologically relevant and thus useful for predictions in other systems. In this model, “steps” do not represent physical steps of animals - rather, they represent decision points [21]. At each step the animal chooses the next spot to go to and travels towards it; the straight section from one decision point to another represents an animal walking in oriented movement towards a specific point. Thus, the unit length for steps represents the perceptual range of animals while traveling inside their patch habitat. This is also why the width band of edge effects was set at 1 unit from the edge. This conception of the units allows us to apply some of the quantitative predictions to other systems. A key result is that for all of these responses, the further from the tip of the shape, the smaller the response, with effective no response past 40% of the perceptual range of the animal. This result occurs over a four-fold range of shape widths (width/height 0.4 to 1.5), and edge reaction functions from strong avoidance to strong attraction. This means that one can apply these predictions to realistic situations, appropriately scaled. For example, Fender's blue butterflies [8] turn towards edges from a distance of 10 m away. Thus one would expect that these butterflies would collect within ∼4m of the tip of edge convexities, and would disperse from within ∼4m of the tip of concavities.

These results also generate predictions for the effects of edge tortuousity. It has been long noted that a higher perimeter:area ratio increases emigration [17], [22], [23], and that thus more tortuous edges should be more permeable. My results suggest that this effect can be magnified by edge shape effects. Assuming a similar number of convex and concave edges, the response to tortuous edges depends on the edge behavior. Animals that avoid edges show a symmetrical response, with similar positive and negative responses to concavities and convexities with a greater positive effect for convexities than a negative effect for concavities (**Figure 5** **C**). Thus emigration should show no predictable response to edge tortuosity. On the other hand, animals that are attracted to edges show an asymmetrical response, with a greater positive effect for convexities than a negative effect for concavities (**Figure 5** **A**). Thus emigration should increase with edge tortuousity when animals are attracted to edges. It would be useful for theoretical and field studies to explicitly test the effects of tortuous edges on permeability.

### Ecological aspects of edge shape

My results showed that simply reacting to edges causes an emergent property of responding to edge geometry. This considerably broadens the scope of edge shape effects because animals need a certain level of cognition to recognize edge geometry; one would expect many more species to recognize and react to edges (for example, [24]–[26]) than to edge shapes. Furthermore, all edges have convexities and concavities at some scales; edge shape effects might very be common.

Thus edge geometry could have important effects on local ecological communities. Edge shapes tend to either concentrate or disperse animals, simply because the animals are either attracted to or avoid edges, with an effect as great as 3 times the normal density. This effect would be felt both up and down the food chain. Here are three examples. First, many small mammals respond to edges [27]; a high proportion of seeds are eaten by small mammals [28] and many predators prey on small mammals. Second, some pollinating bee species (e.g. *Bombus lapidarius* L. bumblebees [29] and honey bees [30]) avoid edges while foraging for pollen inside of patches of flowering plants. Finally, many birds have been shown to respond to edges [25], and many species of plants are primarily dispersed by birds. Thus edge shape could affect pollination, seed predation and dispersal and predator abundance. It would be useful to test these prediction in the field.

Another potential effect of edge shape that more directly affects humans, is on the behavior of large ungulates. Collisions between vehicles and large wildlife (e.g. white-tailed deer (*Odocoileus virginianus*) [31], moose (*Alces alces*) [32], brown bears (*Ursus arctos*) [33]) cause significant damage in both North America and continental Europe. This significantly affects not only vehicles, but some local populations that are in danger of extinction because of vehicle collisions (e.g. Florida panthers (*Puma concolor coryi*) [34] in Florida, black bears (*Ursus americanus*) near urban areas [35], and ocelot (*Leopardus pardalis)* in United States [36]). One factor that affects the likelihood of wildlife-vehicle collisions is the landscape features of the surrounding area [37], [38]. Thus it may be possible to ameliorate the effects of roads on wildlife by manipulating the geometry of habitat edges near roads.

### Future directions

This is a new area of research and my model has only been able to answer a few questions. It would be useful to test these model predictions with experimental and field work, and also to explore general questions. How do edge shapes affect animal movement, edge permeability and emigration? Which types of animals respond most to edge shape? Which properties of animals could be used to predict animal response. These types of studies have been difficult in the past because it has been difficult to track animals with a high enough resolution to identify edge behavior, and with enough data points to have enough edge shape encounters. However in the last ten years GPS devices for remotely tracking animals have increased in capability. For example GPS collars now are as light as 150 grams [39], allowing biologists to track animals as light as 1 kg. Locations can be obtained every 10 seconds as accurately as 2.5 m. This capability in animal tracking should allow us to answer questions about effects of edge geometry on animal movement.
